# Decellularization of porcine skeletal muscle extracellular matrix for the formulation of a matrix hydrogel: a preliminary study

**DOI:** 10.1111/jcmm.12776

**Published:** 2016-01-19

**Authors:** Yuehe Fu, Xuejiao Fan, Chunxiang Tian, Jingcong Luo, Yi Zhang, Li Deng, Tingwu Qin, Qing Lv

**Affiliations:** ^1^ Department of Thyroid and Breast Surgery West China Hospital Sichuan University Chengdu Sichuan China; ^2^ Division of Stem Cell and Tissue Engineering State Key Laboratory of Biotherapy West China Hospital Sichuan University Chengdu Sichuan China

**Keywords:** tissue engineering, extracellular matrix, tissue scaffold, skeletal muscle, hydrogel

## Abstract

Extracellular matrix (ECM) hydrogels are used as scaffolds to facilitate the repair and reconstruction of tissues. This study aimed to optimize the decellularization process of porcine skeletal muscle ECM and to formulate a matrix hydrogel scaffold. Five multi‐step methods (methods A–E) were used to generate acellular ECM from porcine skeletal muscle [rinsing in SDS, trypsin, ethylenediaminetetraacetic acid (EDTA), Triton X‐100 and/or sodium deoxycholate at 4–37°C]. The resulting ECM was evaluated using haematoxylin and eosin, 4‐6‐diamidino‐2‐phenylindole (DAPI) staining, and DNA quantification. Acellular matrix was dissolved in pepsin and gelled at 37°C. Hydrogel response to temperature was observed *in vivo* and *in vitro*. ECM components were assessed by Masson, Sirius red, and alcian blue staining, and total protein content. Acellular porcine skeletal muscle exhibited a uniform translucent white appearance. No intact nuclear residue was detected by haematoxylin and eosin staining, while DAPI staining showed a few nuclei in the matrixes produced by methods B, C, and D. Method A generated a gel that was too thin for gelation. However, the matrix obtained by rinsing in 0.2% trypsin/0.1% EDTA, 0.5% Triton X‐100, and 1% Triton X‐100/0.2% sodium deoxycholate was nuclei‐free and produced a viscous solution that formed a structurally stable white jelly‐like hydrogel. The residual DNA content of this solution was 49.37 ± 0.72 ng/mg, significantly less than in fresh skeletal muscle, and decreased to 19.22 ± 0.85 ng/mg after gelation (*P* < 0.05). The acellular matrix was rich in collagen and glycosaminoglycan, with a total protein concentration of 64.8 ± 6.9%. An acellular ECM hydrogel from porcine skeletal muscle was efficiently produced.

## Introduction

Decellularized extracellular matrix (ECM) has been used for over 20 years to help the repair and reconstruction of tissues. Acellular ECM can provide structure, native tissue cell adhesion proteins, growth factors, and glycosaminoglycans to direct site‐appropriate remodelling in the host 1, 2, 3. However, antigenic cellular proteins, lipids and nucleic acids that may induce an inflammatory reaction and the eventual rejection of the implants must first be removed from donor tissues by removing the cells 1.

Hydrogels are a class of three‐dimensional (3D) scaffold material that can maintain three‐dimensional structure, absorb and retain large volumes of water 4, and exhibit a plasticity similar to that of the microstructure of native ECM 5, 6, 7. Hydrogel can be transplanted by minimally invasive injection of liquid hydrogel, which then solidifies at the site of injection. Therefore, the acellular hydrogel matrix is a popular scaffold material 4, 8, 9, 10. Hydrogels derived from the acellular matrix of derma, fat, small intestinal submucosa, bladder, myocardium, pericardium, brain, placenta, liver and tendons have been widely used for the repair and reconstruction of soft tissues, bone, cartilage, tendon, oesophagus, myocardium and other tissues and organs 11, 12, 13, 14, 15, 16, 17, 18, 19.

Decellularization of ECM can be achieved by a variety of techniques. These processes generally involve physical methods (grinding into a powder, striking, shaking, pressing or repeated freezing and thawing), chemical reagents (detergents, organic solvents, low/high permeability saline solution), and/or biological reagents (enzymes and coenzymes including lipase and trypsin) to dissolve or digest cellular components 1. The low permeability method involves the application of osmotic pressure so that cytoplasm expansion leads to cell membrane swelling and cracking, therefore releasing the cytoplasm proteins, but the cell residual materials cannot be removed completely 1. Sequential combination of these techniques is usually required to achieve the complete removal of cells. Decellularization techniques have been established for pig, rat and human tissues including the derma, fat, small intestinal sub‐mucosa, bladder, myocardium, pericardium, brain, placenta, liver and tendons, but the preparation of acellular skeletal muscle remains technically challenging and few studies have reported the successful generation of a hydrogel derived from skeletal muscle acellular matrix 1, 20.

The blood vessel density of porcine skeletal muscle is high, and the tissue is rich in growth factors, collagen, laminin and other active substances 21, 22. Furthermore, porcine skeletal muscle is readily available and thus suitable for large‐scale experimental studies and clinical practice. In implants of xenogeneic origin, maximal decellularization is required to minimize the risk of adverse immune responses. This study sought to define a procedure for the thorough decellularization of porcine skeletal muscle to generate an acellular ECM for use as a hydrogel. While there are a number of different decellularization methods, they are generally time‐consuming and include the repeated and long‐term use of chemical reagents and enzymes that tend to damage the components of the ECM and structure of the gel, impairing hydrogel formation 1, 20. Therefore, we sought to modify and improve upon methods of porcine skeletal muscle decellularization. We pursued five schemes that were developed according to previously described decellularization methods 8, 11, 16, and the characteristics of the skeletal muscle itself. The methods that were most convenient and achieved the complete removal of antigen and maximum preservation of ECM were applied to the development of a porcine skeletal muscle acellular matrix hydrogel, to generate a novel tissue engineering scaffold material.

## Materials and methods

### Animals

Fresh skeletal muscle tissue was obtained from healthy adult pigs after quarantining them in a slaughterhouse. Twenty‐four healthy adult male SD rats (8–9 weeks old, 300–350 g) were purchased from Chengdu Dashuo Biological Technology Co., Ltd. (Tianfu Life Science Park, Chengdu, Sichuan, China) and bred at the clean grade West China Medical Experimental Animal Center of Sichuan University. Animals were maintained between 18 and 26°C, in 40–70% humidity, <20 PPM of ammonia, <85 dB of noise and ventilation 8–12 times/hour with an indoor air velocity of 0.1–0.2 m/sec. and a 12‐hr light/dark cycle. Sufficient feed was added twice weekly to ensure dry and fresh feed. Drinking water (pH 2.5–2.8) was replaced 2–3 times per week. Animal nests were replaced 1–2 times per week. The study was approved by the Animal Care and Use Committee of Sichuan University.

### Preparation of porcine skeletal muscle acellular matrix

Fresh skeletal muscle was collected from healthy adult pigs within half an hour of sacrifice. The fascia was removed and the muscle was cut into small pieces (1–2 mm^3^). Samples were rinsed with deionized water to remove blood and impurities.

The decellularization process involved either: shaking in 1% SDS (Sigma‐Aldrich, St Louis, MO, USA) at room temperature (RT) for 72 hrs (scheme A); shaking in 1% SDS at RT for 24 hrs and then shaking in 0.2% sodium deoxycholate (Sigma‐Aldrich) at RT for 48 hrs (scheme B); shaking in 1% SDS at RT for 24 hrs, followed by shaking in 1% Triton X‐100 (import repacking) at RT for 48 hrs (scheme C); shaking in enzyme digestion solution [0.2% trypsin, (GIBCO, Invitrogen Inc., Carlsbad, CA, USA)/0.1% ethylenediaminetetraacetic acid (EDTA; Sigma‐Aldrich)] at 37°C for 1 hr, in 0.5% Triton X‐100 at 37°C overnight, and then in 1% Triton X‐100/0.2% sodium deoxycholate at RT for 6–8 hrs (scheme D); or shaking in enzyme digestion solution (0.2% trypsin/0.1% EDTA) at RT for 3 hrs, in 0.5% Triton X‐100 at 4°C overnight, and then in 1% Triton X‐100/0.2% sodium deoxycholate at RT for 6–8 hrs (scheme E).

Samples were rinsed in deionized water and the white matrix was milled into a coarse powder by low temperature ball milling (Retsche, Haan, Düsseldorf, Germany). The powder was shaken in isopropanol at RT overnight and rinsed in deionized water until the isopropanol smell was removed.

Residual nuclear materials were removed by shaking in 5 × 10^7^ U/l DNase‐I (Roche Molecular Systems, Pleasanton, CA, USA) and 1 × 10^6^ U/l RNase (Roche Molecular Systems) at RT overnight. The residual reagents were removed by repeated rinsing in triple‐distilled water and centrifugation. The obtained white matrix was frozen‐dried, sealed and stored at 4°C.

### Histological analysis

The fresh skeletal muscle and acellular white matrix were fixed in 4% paraformaldehyde for 24 hrs, rinsed with water overnight, dehydrated in graded alcohol, embedded in paraffin and sliced in 4–6 μm serial sections (Leica Microsystems, Wetzlar, Germany).

Haematoxylin and eosin and DAPI (Vector Laboratories, Burlingame, CA, USA) staining were performed after routine antigen retrieval, dewaxing and rehydrating. After conventional haematoxylin and eosin staining, sections were sealed with neutral gum and assessed by light microscopy (Olympus, Tokyo, Japan). Each section was covered with 50 μl of DAPI (0.1 μg/ml in PBS) and stored in the dark. Staining was assessed by an E600 fluorescence microscope (Nikon, Tokyo, Japan) after 5 min. Serial sections (4–6 μm) were stained with Sirius red, alcian blue, and Masson trichrome to assess the collagen and glycoprotein composition. The sections were observed by light microscopy.

### Preparation of porcine skeletal muscle acellular matrix temperature‐sensitive hydrogel and assessment of thermo‐sensitive properties

**Figure 1:**
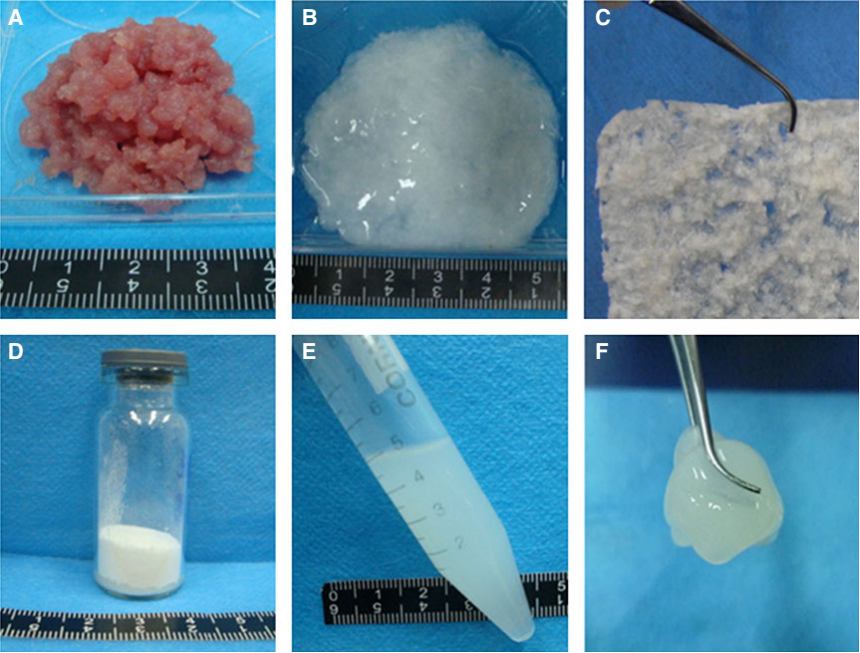
Preparation of porcine skeletal muscle acellular matrix temperature‐sensitive hydrogel. (**A**) Before decellularization. (**B**) After decellularization. (**C**) Frozen‐dried material. (**D**) Baked powder. (**E**) Dissolution. (**F**) Gelation.

As previously described 11, 12, 10 mg of porcine skeletal muscle acellular matrix powder was added to 900 μl of pepsin (Sigma‐Aldrich) solution (0.01 M HCl, pepsin: ECM = 1:10) and incubated under shaking at RT for 48 hrs until dissolved. On an ice bath, 9 μl of 1 M NaOH were dripped into the viscous liquid to stop the digestion; 10× PBS was added and evenly mixed, and the mixture was incubated at 37°C for gelation for 30 min., 2 hrs, and 24 hrs *in vitro* (Figs 1 and 4).

The dissolved and neutralized matrix solution was stored at 4°C in an ice bath. The matrix solution (800 μl) was placed in a water bath at 37°C gelation was assessed every 10 min. Twenty‐four SD rats were weighed. The abdominal hair was shaved and the skin was sterilized with iodine and alcohol. The matrix solution (800 μl) was then rapidly subcutaneously injected using a 25G 1‐ml syringe. Each rat was symmetrically injected at four points within about 1 min. Three rats were killed immediately, and the remaining rats were returned to their cages. Three more rats were killed every 10 min. for 1 hr, *i.e*. at seven time‐points. Three rats were killed 24 hrs after gelation. The abdominal skin was incised, the subcutaneous tissues were separated layer by layer, and the injected material was excised with the skin overlaying it.

### Quantitative detection of DNA and re‐verification of the acellular scheme

Fresh skeletal muscle, acellular matrix and gel material were frozen‐dried. Samples (10 mg) were digested in 500 μl of proteinase K solution (100 μg/ml; Sigma‐Aldrich) at 60°C for 48 hrs. After centrifugation at 10,000 × g and 4°C for 10 min., the supernatant was collected and added to an equal volume of phenol‐chloroform (V/V = 1:1). After shaking for 10 min., the mixture was centrifuged at 10,000 × g and 4°C for 30 min. The supernatant was mixed with 3 M sodium acetate (sodium acetate: supernatant = 1:1, V/V) and ethanol (ethanol: sodium acetate + supernatant = 2.5:1, V/V) was then added. The solution was mixed and incubated at 4°C overnight to precipitate the DNA (filamentous material). The solution was then centrifuged at 10,000 × g for 10 min. The precipitate was dried and the DNA content was measured with a Quant‐iTTM PicoGreen^®^ dsDNA Assay Kit (Invitrogen Inc.), as previously described and according to the manufacturer's instructions 23.

### Assessment of porcine skeletal muscle acellular matrix structure and protein content

The Bicinchoninic Acid (BCA) protein detection kit was used according to the manufacturer's instructions (Sigma‐Aldrich). The ECM solution was dissolved to 0.05% in PBS and 20 μl were added to five wells of a 96‐well plate. BCA (200 μl) was added in each well and the plate was incubated on a shaker at 37°C for 30 min. After cooling to RT, the absorbance was measured at 562 nm using a microplate reader (Bio‐Rad, Hercules, CA, USA). Protein concentration was calculated using a standard curve.

### Scanning electron microscopy

After washing with phosphate buffer, the hydrogel samples were dehydrated and dried under vacuum (Heto GmbH, Martin Christ Gefriertrocknungsanlagen GmbH, Osterode Germany). Then the dried gel was cut and the cross‐section was sprayed with platin/palladium. Gel cross‐sections were studied by scanning electron microscopy using a Zeiss DSM 962 (Inspect‐F; FEI, Hillsboro, Oregon, USA) scanning electron microscope.

### Mechanical analysis

Syringes (10 ml) were filled with pre‐gelation solutions at 1.0% ECM, 1.2% ECM, and 1.4% ECM in an ice bath. The syringes were placed in an incubator at 37°C. Three cylindrical pieces of 9–10 mm in length were made for each concentration by pushing the plunger of the syringes. A Vernier caliper was used to measure the exact length and diameter of each piece. A biomechanical dynamic stress testing machine was used to test the mechanical properties of the gels. The compressing experiment was performed at a 2‐mm/min compression speed, until gel rupture. According to the acquisition system of the testing machine, compressive strength and compressive deformation were used to calculate the elastic modulus and stiffness of the sample.

### Cell Counting Kit‐8 assay for cytocompatibility testing

The viability of mouse NIH3T3 cells (American Type Culture Collection, Manassas, VA, USA) cultured on the 1% ECM hydrogel was assessed by a Cell Counting Kit‐8 assay (CCK‐8) according to the manufacturer's instructions (#26992; Sigma‐Aldrich) and commercial collagen I hydrogel composed of mouse tail collagen I (Millipore Corp., Billerica, MA, USA) as control. NIH3T3 cells were seeded in a 96‐well plate at a density of 8000 cells/well. The absorbance was measured at 450 nm after culturing for 1, 2, 3, and 4 days.

### Statistical analysis

SPSS 18.0 (IBM, Armonk, NY, USA) was used to analyse data. Data are expressed as mean ± S.D. and were analysed using the Student's *t*‐test. Two‐tailed *P*‐values <0.05 were considered statistically significant.

## Results

### Preliminary evaluation of the acellular matrix

**Figure 2:**
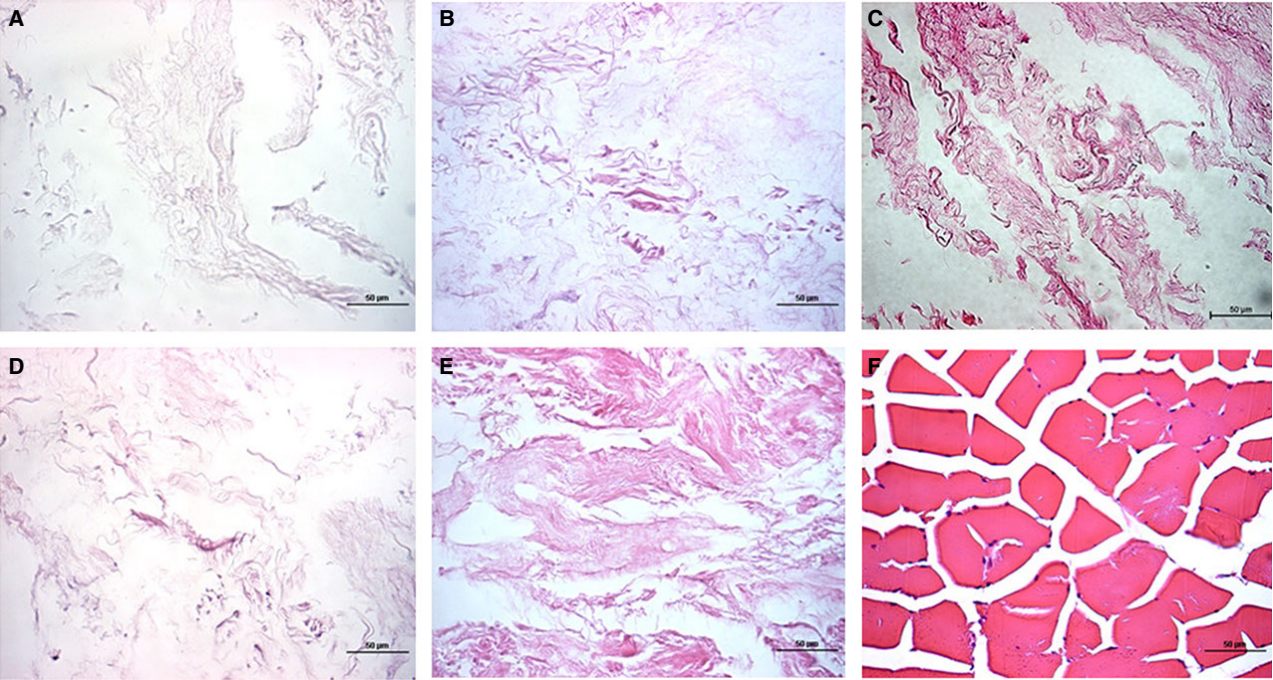
Haematoxylin and eosin staining of porcine skeletal muscle before and after decellularization. (**A**–**E**) Decellularization methods **A**–**E** respectively. (**F**) Fresh porcine skeletal muscle. Samples were stained with haematoxylin and eosin (×400).

**Figure 3:**
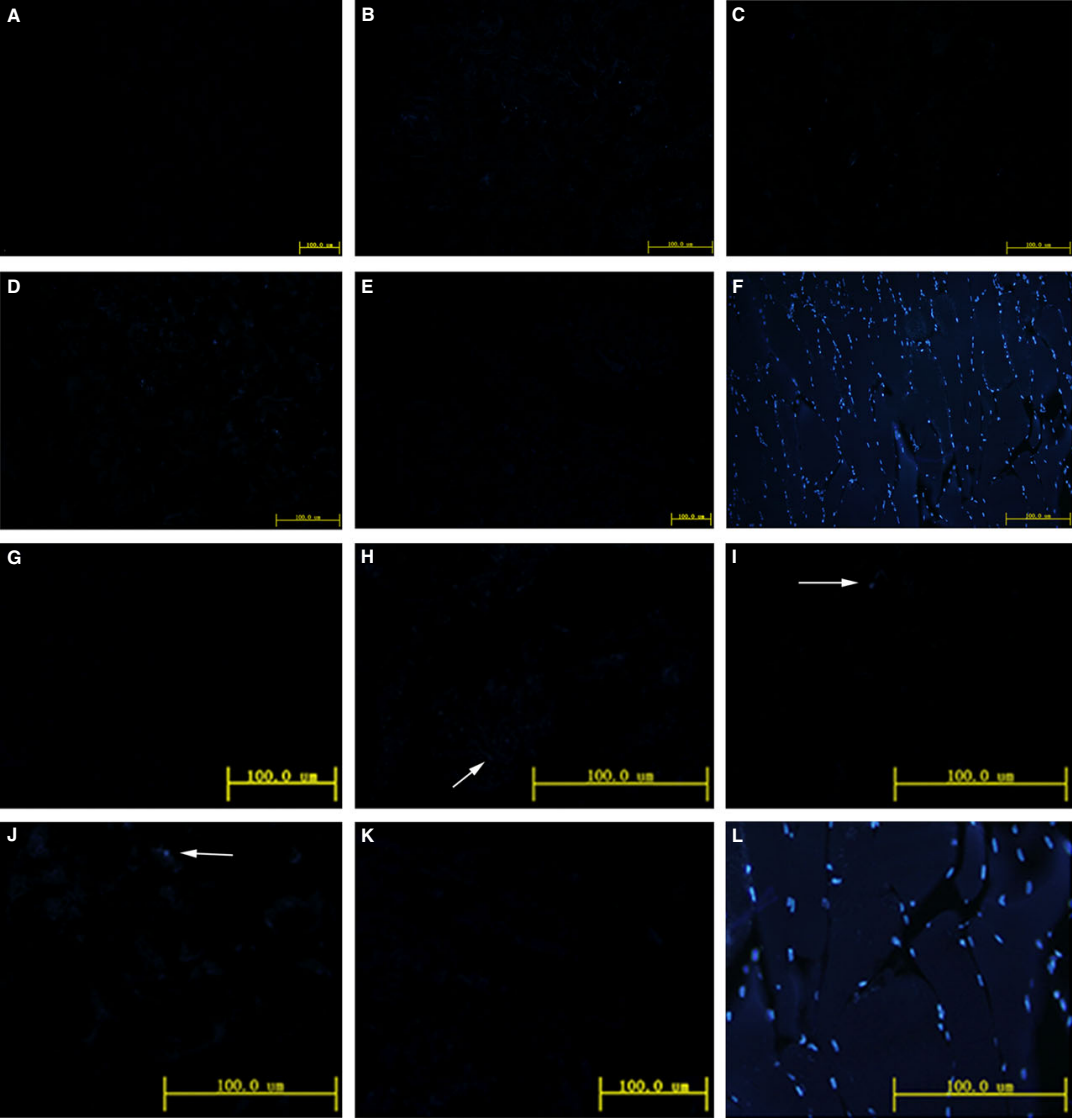
DAPI staining of porcine skeletal muscle before and after decellularization. (**A**–**E**) Decellularization methods **A**–**E** respectively. (**F**) Fresh porcine skeletal muscle. Samples were stained with DAPI (×200). (**G**–**L**) The high magnification of **A**–**F** respectively.

The fresh porcine skeletal muscle was pink, but after decellularization, the matrix was a glossy translucent uniform milky white (Fig. 1A and B). Haematoxylin and eosin staining showed that the cell membrane structure was intact after decellularization, and hematoxylin‐stained nuclei were clearly visible only in fresh skeletal muscle (Fig. 2F). DAPI staining highlighted the nuclei of normal muscle cells (Fig. 3F). After decellularization using methods A and E, almost no nuclei were observed (Fig. 3A (G), E (K)), but some nuclei remained after decellularization using methods B, C and D (Fig. 3B–D (H‐J)). Therefore, methods A and E were used for the subsequent experiments.

### Preparation of porcine skeletal muscle acellular matrix temperature‐sensitive hydrogel and assessment of its thermo‐sensitive properties

**Figure 4:**
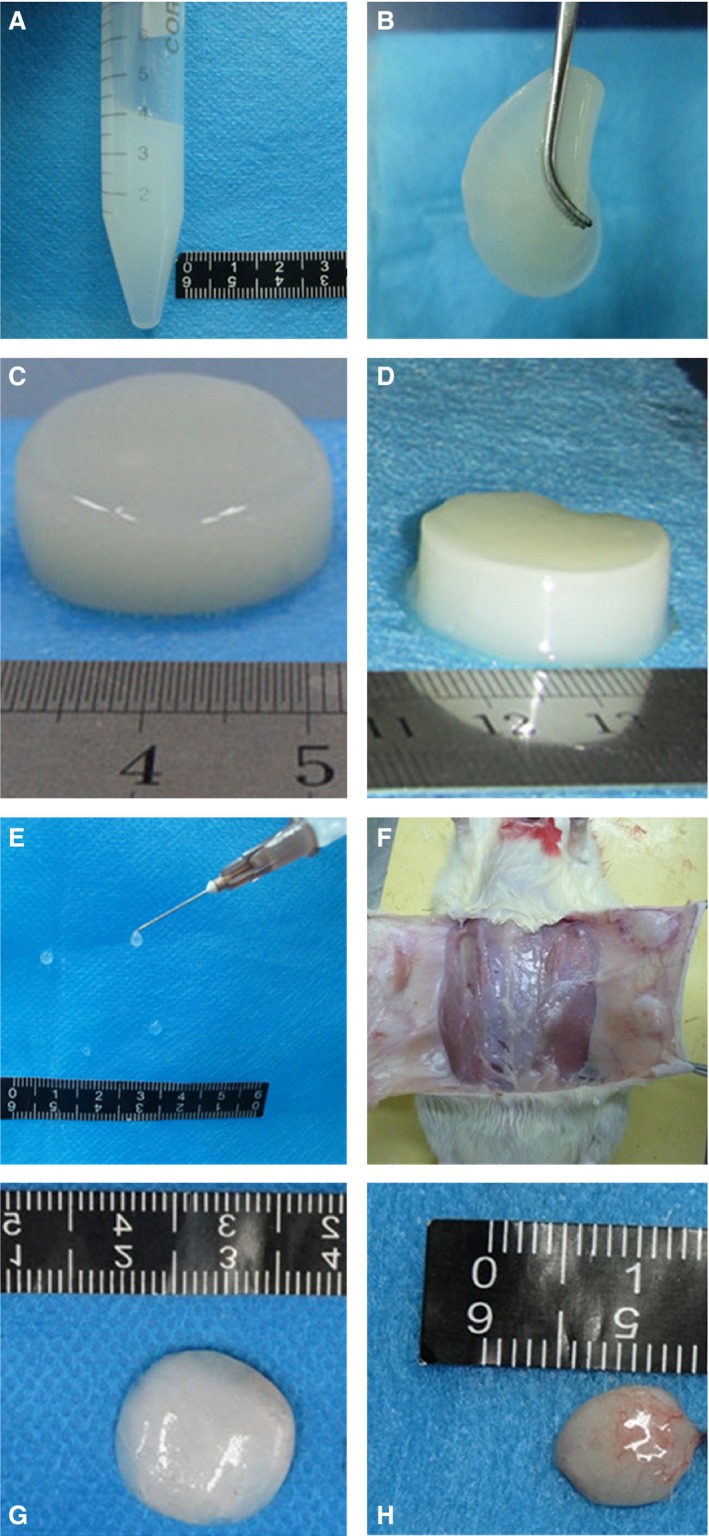
*In vivo* and *in vitro* gelation of porcine skeletal muscle acellular matrix hydrogel. Gelation of acellular matrix hydrogel *in vitro* (**A**–**D**) and *in vivo* (**E**–**H**). (**A**) Acellular matrix hydrogel maintains a liquid state at low temperatures, ideal for administration using a syringe. (**B**) Acellular matrix hydrogel solidifies after 30 min. at 37°C, but remains malleable. (**C**) After 2 hrs of incubation at 37°C, the hydrogel can easily be manipulated into any shapes using different moulds (*e.g*. a cylinder) and maintain a stable structure. (**D**) The hydrogel after 24 hrs of incubation at 37°C. (**E**) Acellular matrix hydrogel in the liquid state can be administered by a syringe. (**F**) Acellular matrix hydrogel solidified at four positions within the rat abdomen 30 min. after injection. (**G**) Solidified acellular matrix hydrogel 30 min. after injection was separated from rat tissues. (**H**) Solidified acellular matrix hydrogel 24 hrs after injection was separated from rat tissues.

Dissolution and gelation of the acellular matrices obtained by methods A and E were assessed in parallel. The dissolved matrix obtained by method A was thin, and did not jellify at 37°C. The dissolved matrix obtained by method E was more viscous and more transparent (Fig. 4A). The hydrogel was gelled after 30 min. at 37°C (Fig. 4B). After 2 hrs, the white jelly hydrogel had a stable structure (Fig. 4C). Therefore, the hydrogel produced by method E was used in subsequent experiments.

Hydrogel (800 μl) was injected subcutaneously in the abdomen of rats. Rats were killed every 10 min. and the hydrogel was excised and studied. The hydrogel was gelled at 30 min. (Fig. 4E and F). To evaluate the mid‐term stability of the hydrogel *in vitro* and *in vivo*, the hydrogel was evaluated 24 hrs after injection into rats. Figure 4 shows that the hydrogel *in vitro* after 24 hrs has sharp edges and good shape (Fig. 4D), indicating that it was more stable than hydrogel after 2 hrs *in vitro* (Fig. 4C). In addition, *in vivo* data show that the volume of hydrogel *in vivo* after 24 hrs (Fig. 4H) was much smaller than the hydrogel after 2 hrs *in vivo* (Fig. 4G) since most water in the hydrogel was probably absorbed by neighbouring tissues. Compared to the 2‐hr hydrogel, the structure of the 24‐hr hydrogel was more compact, the elasticity was higher, and the stability was higher.

### DNA quantitative assay

**Figure 5:**
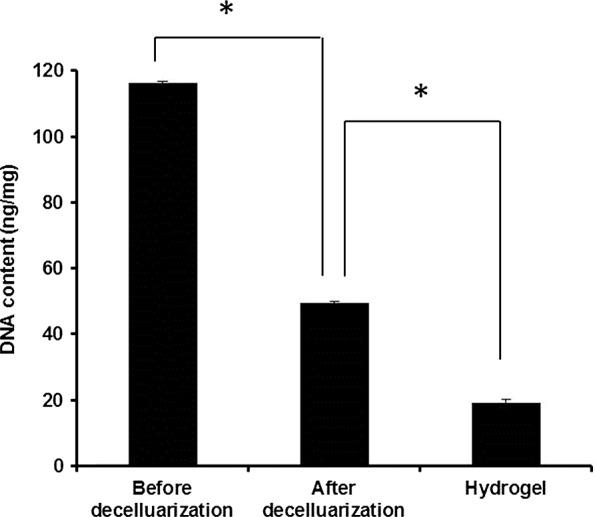
DNA content of skeletal muscle, ECM, and hydrogel. DNA content of skeletal muscle, ECM, and hydrogel was assessed by the Quant‐iTTM PicoGreen^®^ ds DNA Assay Kit. *P < 0.05 (*n* = 3).

The DNA content of the fresh porcine skeletal muscle was 116.16 ± 0.61 ng/mg and after decellularization by method E, it was significantly reduced to 49.37 ± 0.72 ng/mg (*P* < 0.05, *n* = 3; Fig. 5). After being frozen‐dried and digested by pepsin, the residual DNA content of the gel was further significantly reduced to 19.22 ± 0.85 ng/mg (*P* < 0.05, *n* = 3).

### Composition of the acellular matrix

**Figure 6:**
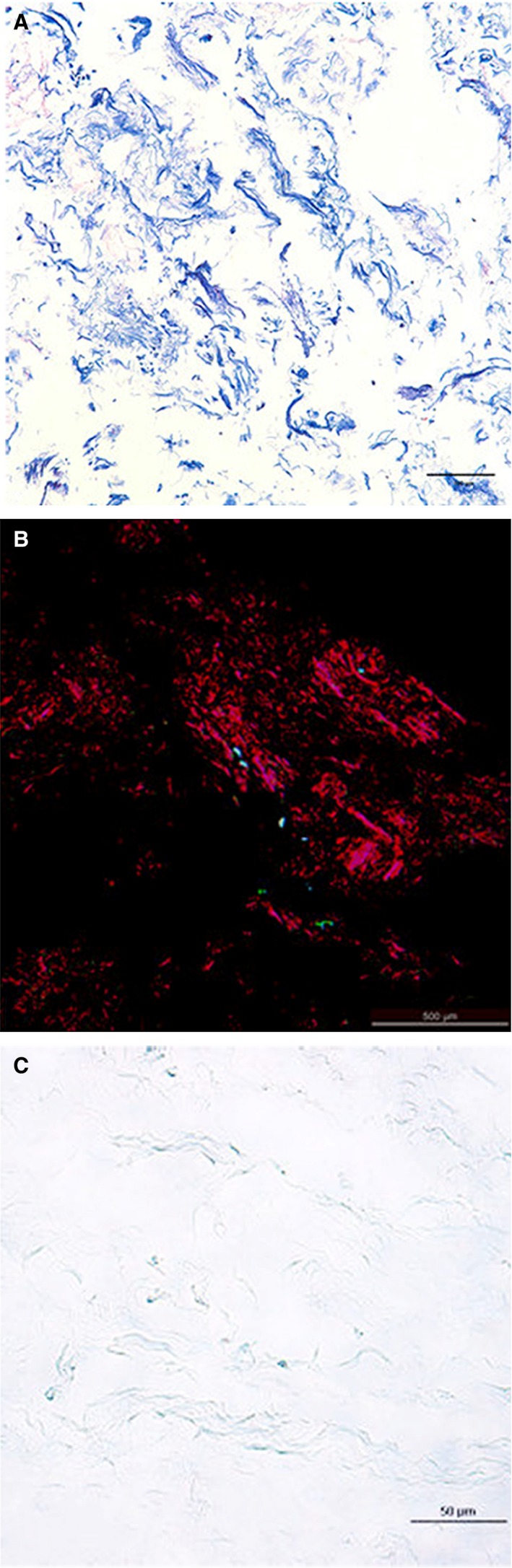
Porcine skeletal muscle collagen structure. (**A**) Masson staining (×200). (**B**) Sirius red staining (×40). (**C**) Alcian blue staining (×400).

Histological analysis indicated that porcine skeletal muscle acellular matrix contained closely spaced Masson‐stained collagen fibres (Fig. 6A), large densely packed Sirius red stained type I collagen fibres, staggered with some type III collagen fibres and type IV collagen fibres (Fig. 6B). Flocculent and ribbon‐like acidic glycoproteins were visualized by Alcian blue staining, indicating sulphated glycosaminoglycan (Fig. 6C).

**Figure 7:**
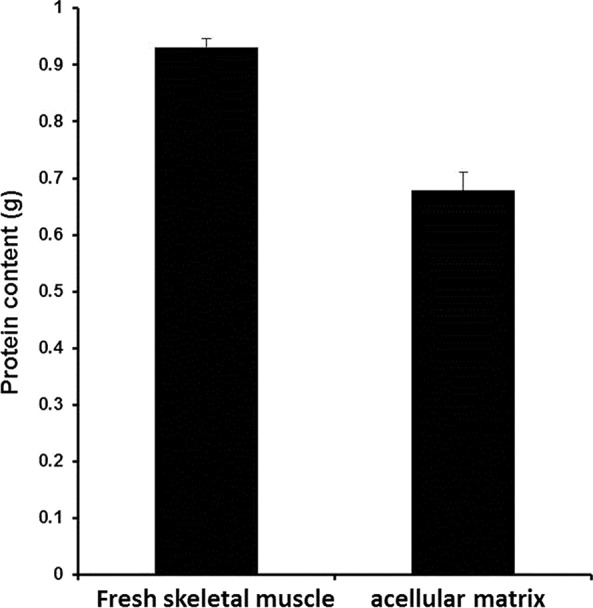
Protein content of skeletal muscle and decellularized ECM. Protein content of skeletal muscle and decellularized (ECM) was assessed using a BCA kit. *P < 0.05 (*n* = 3).

**Figure 8:**
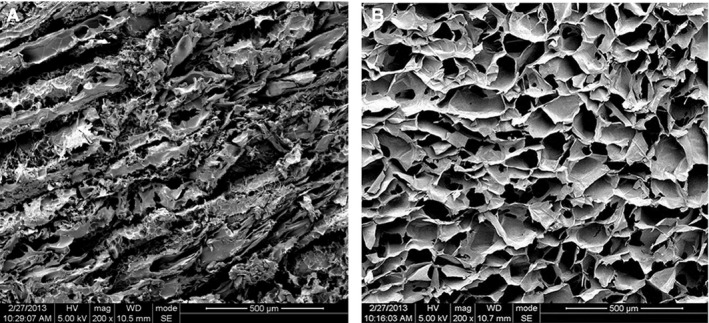
Scanning electron microscopy. Micrograph of a cross‐section of fresh skeletal muscle tissue (**A**) and hydrogel derived from decellularized porcine skeletal muscle matrix (**B**).

The protein content of porcine muscle tissue after decellularization was 64.8 ± 6.9%, *i.e*. 1 g of dry ECM contained 0.648 ± 0.069 g of proteins (Fig. 7). Scanning electron microscopy demonstrated that skeletal muscle tissue contained muscle fibres, while decellularized hydrogel matrix was composed of a porous structure with various pore sizes (Fig. 8).

### Mechanical analysis

The mechanical properties of different ECM concentrations and the statistical difference among the groups are provided in Table 1. ECM at 1.4% had a significantly higher maximum load than 1.0% and 1.2%, as well as better compressive strength, elastic modulus and stiffness. There was no difference in compressive deformation.

**Table 1:** Mechanical properties of ECM hydrogel at different concentrations (*n* = 3)

ECM concentration	Mechanical properties				
	Maximum load (*N*)	Compressive strength (kPa)	Compressive deformation (%)	Elastic modulus (MPa)	Stiffness (*N*/mm)
1.0%	0.769 ± 0.083	4.075 ± 0.441	56.244 ± 9.511	0.100 ± 0.000	0.214 ± 0.363
1.2%	1.105 ± 0.213	5.856 ± 1.128	48.278 ± 6.391	0.200 ± 0.000	0.500 ± 0.077
1.4%	2.635 ± 0.390	13.962 ± 2.068	53.460 ± 10.910	0.267 ± 0.058	0.574 ± 0.138
*P*‐value	<0.001	<0.001	0.585	0.003	0.007

### Cytocompatibility

**Figure 9:**
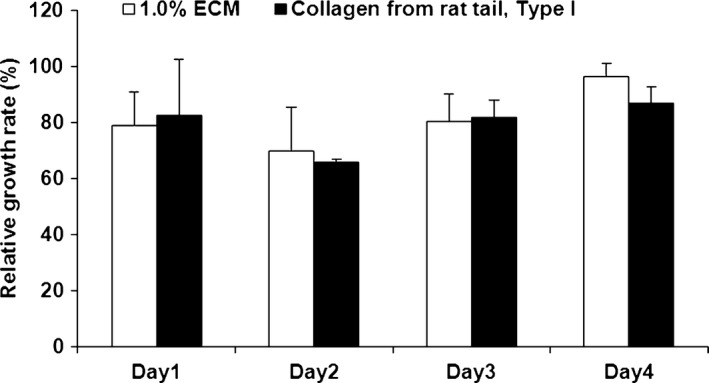
CCK‐8 assays. Relative growth rate of NIH3T3 cells after being culturing for 4 days on porcine skeletal muscle decellularized ECM hydrogel and collagen I hydrogel as the control group. Results are shown as mean ± S.D.; *n* = 3.

The growth of NIH3T3 cells cultured on both porcine skeletal acellularized ECM hydrogel and commercial mouse tail collagen I hydrogel was good and there was no significant difference in the relative growth rate between the two groups over 4 days (*P* > 0.05, *n* = 5; Fig. 9).

## Discussion

Extracellular matrix hydrogels can act as scaffolds to facilitate the repair and reconstruction of tissues. We sought to develop an optimized technique for decellularization of porcine skeletal muscle ECM and to formulate a matrix hydrogel scaffold. Indeed, hydrogel materials derived from porcine skeletal muscle must be thoroughly decellularized to ensure biological safety. However, the chemical reagents conventionally used to decellularize tissues can damage the components and structure of the ECM 1, 20. We sought to establish a simple and efficient method to achieve complete removal of antigens, with maximal preservation of ECM to obtain an improved tissue‐engineered scaffold.

We tried five protocols for decellularization, each involving incubation of the porcine skeletal tissue in a series of solutions. SDS is a commonly used anionic detergent that denatures proteins, making them less soluble. SDS has previously been used successfully in decellularization 1, 20. Sodium deoxycholate (an ionic detergent that can dissolve membrane proteins and cleave cells) and Triton X‐100 (a nonionic detergent that can disrupt tissues) were tried, and they had a gentler effect on the ECM compared to SDS 1. Trypsin was previously reported to gently and effectively decellularize ECM, and the extent of the damage to the ECM was associated with the reaction time and temperature 13. The degreasing agent isopropanol has also been used 1 in addition to DNAase and RNAase, which catalyse the hydrolysis of DNA and RNA 24.

Although decellularized muscle tissue has been used in tissue engineering, the use of porcine skeletal muscle matrix hydrogels has rarely been reported. In 2012, DeQuach *et al*. 2 successfully prepared a porcine skeletal muscle matrix hydrogel and used this hydrogel in an animal model of lower limb ischaemia. They produced decellularized skeletal muscle by washing in deionized water, incubating 4–5 days in 1% SDS, and incubating overnight in deionized water to remove residual SDS 25. We sought to improve this method, and developed a quicker technique for the generation of acellular ECM from porcine skeletal muscle. In this study, pig skeletal muscle was ground into 1–2‐mm^3^ pieces to increase the action area of isopropanol and other reagents and the decellularized ECM was finally ground into a powder to improve the removal of residual reagents. The final decellularized ECM product was rinsed in triple‐distilled water and centrifuged 8–10 times until no residual isopropanol odour could be detected.

Although no clear and unified standards for the evaluation of decellurization have been established, we aimed to fulfil the following minimum requirements 1: (*i*) no nuclei were detected by DAPI or haematoxylin and eosin staining of sections; (*ii*) the dry weight of double‐stranded DNA content was <50 ng/mg and (*iii*) no DNA fragments over 200 base pairs could be detected. The products of all five decellularization methods tried in this study contained no intact cells; however, DAPI staining revealed the presence of residual nuclei in the products of methods B, C and D. Therefore, only the products of methods A and E were used for subsequent experiments.

As previously reported, pepsin was used to digest the matrix for up to 48 hrs 11, 12. Pepsin breaks the peptide bonds at specific sites in the non‐helical region of the collagen chain to remove the non‐helical immunogenic region of collagen 3, 25. The pH was then adjusted and the matrix was incubated at 37°C. The matrix produced by method E eventually formed a three‐dimensional hydrogel scaffold material, while the matrix produced by method A was unable to form a hydrogel. Method A used SDS, which has been reported to damage the ECM and collagen ultrastructure, whereas method E used sodium deoxycholate and Triton X‐100, which are less aggressive than SDS, suggesting that the failure of the product of method A to jellify may be attributed to damage to the ECM caused by SDS 1. Method E also generated a decellularized product in 4 days, whereas the method reported by DeQuach *et al*. 2 required 6 days, indicating that our novel decellularization method may be faster, cheaper and induce less damage to the ECM. Nevertheless, the method by DeQuach *et al*. 2 achieved good results *in vivo* in restoring muscle mass after ischaemia. Another method by Wolf *et al*. 8 also achieved good results.

Dissolved in the pepsin solution, the decellularized product of method E formed a viscous fluid within 48 hrs. After neutralizing the pH at a low temperature (4°C ice bath), the viscosity of the solution increased, and at 37°C the hydrogel gradually gelled. Fluid hydrogel at 4°C was injected into rats and gradually gelled at animal body temperature within 30 min., consistent with the ideal properties of a biological scaffold. Similar results were observed in previous studies 2, 8.

The matrix and hydrogel produced by method E contained only 49.37 ± 0.72 ng/mg and 19.22 ± 0.85 ng/mg of residual DNA, which meet the second standard of minimum requirements aforementioned. In this study, DNA was quantified, but the length of the fragments was not measured to comply with criterion 3, aforementioned. Additional work is necessary to address this point.

Skeletal muscle ECM contains collagen, and the collagen arrangement depends upon glycosaminoglycans and other support macromolecules 26, 27. The decellularized porcine skeletal muscle was rich in collagen and glycosaminoglycan. The total protein content of the acellular matrix was 64.8 ± 6.9%, representing 0.648 ± 0.069 g of protein per dry gram. The protein concentration of the hydrogel solution was 1–1.4%, representing 6.48 ± 0.69 to 9.07 ± 0.97 mg/ml, which was similar to the protein content reported for previously described human, pig, mouse and rat hydrogels (about 4–10 mg/ml) 28.

The mechanical properties of the scaffold materials are very important for the support and resistance to the surrounding tissues after implantation 29. The repair of different injured tissues requires different mechanical properties of the scaffolds. The mechanical state of the injectable hydrogel is especially important for the repair and reconstruction of soft tissues 30, but the key weakness of biological sources of hydrogel is the low mechanical strength and lack of stability. This study showed that the mechanical parameters of the gel were improved by increasing the ECM concentration and that the maximum load, compressive strength, elastic modulus and stiffness of the hydrogel were increased with increasing ECM concentration. Therefore, the mechanical properties of the gel can be adjusted by different ECM concentrations, and the properties of the scaffolds may be adjusted according to the tissue to repair. In addition, the ultrastructure of the gel was comparable to another hydrogel previously published 2.

The biocompatibility of scaffold materials must be evaluated *in vitro* before implantation. The cytotoxicity of the materials is an important indicator of the biocompatibility of the materials. Indeed, lower toxicity will lead to less important inflammatory reactions in the host body, resulting in better repair. The cytotoxicity of the material is mainly related to the immunogenicity of the material (*i.e*. the degree of decellularization) and the toxicity of the chemical residues from the treatment. To verify the biological safety of the material, NIH3T3 cells were seeded on the hydrogel made of porcine skeletal muscle decellularized ECM. The results showed that the cell relative proliferation rates of the experimental and control groups were comparable. Therefore, the porcine skeletal muscle decellularized ECM hydrogel obtained using method E had a good *in vitro* cytocompatibility, but we did not assess the mitogenic activity of the ECM matrix, as previously shown by DeQuach *et al*. 2.

Advantages of this protocol are that it is fast and that the mechanical properties of the hydrogel can be adjusted, which was not explored in previous studies 2, 8. Nevertheless, it still requires additional studies before it can be tested in a clinical trial. Importantly, mitogenic and neovascularization properties need to be addressed. Future studies will have to be conducted to assess the exact applicability of this hydrogel, but the fact that its properties can be adjusted will allow its use for the repair of various tissues.

In conclusion, this study established a rapid method for porcine skeletal muscle decellularization, which generated an ECM hydrogel that solidifies *in vivo* and was free of cell components, but retained a rich matrix composition. This hydrogel can form a micro three‐dimensional structure. The mechanical properties of the gel can be adjusted by adjusting the concentration of ECM, which could be used for repair and reconstruction of different tissues. In addition, good biocompatibility makes it promising to be a good tissue scaffold for tissue repair, which has a good application prospect.

## Conflicts of interest

The authors confirm that there are no conflicts of interest.

## Author contribution

YHF performed the research, analysed the data, and wrote the paper. XJF and CXT performed the research and analysed the data. JCL, YZ, LD and TWQ analysed and interpreted the data. QL designed the research study and provided the critical revision. All authors read and approved the final manuscript.
